# Metal accumulation in paired colon cancer and adjacent tissues and its relationship with genotoxic and epigenetic biomarkers

**DOI:** 10.3389/fpubh.2026.1801314

**Published:** 2026-04-14

**Authors:** Iman Al-Saleh, Ghofran Al-Qudaihi, Reem Al-Rouqi, Nujud Alrushud, Hissah Alnuwaysir, Samar Alhomoud, Alaa Abdul Jabbar, Luai Ashari, Raha Alahmadi, Hussain Alkhdhur, Salah Addin Falah, Hadeel Almanea, Hussah Alhussaini, Anmar Semilan, Rasha Althebaity, Saud Alharbi

**Affiliations:** 1 Environmental Health Program, King Faisal Specialist Hospital and Research Centre, Riyadh, Saudi Arabia; 2 Surgical Oncology Department, King Faisal Specialist Hospital and Research Centre, Riyadh, Saudi Arabia; 3 Anatomic Pathology Department, King Faisal Specialist Hospital and Research Centre, Riyadh, Saudi Arabia

**Keywords:** colon cancer, comet assay, environmental exposure, global DNA methylation, tissue–blood biomarkers, trace metals

## Abstract

**Background:**

Environmental exposure to toxic and trace metals has been implicated in colorectal carcinogenesis through oxidative DNA damage and epigenetic dysregulation. However, human evidence linking tissue-level metal accumulation with epigenetic and genotoxic biomarkers remains limited, particularly in studies using paired tumor and adjacent non-cancerous colon tissues.

**Objectives:**

This study aimed to (i) characterize concentrations of selected toxic and trace metals in paired cancerous and non-cancerous colon tissues, (ii) examine associations between tissue metal concentrations and global DNA methylation in colon tissue, and (iii) evaluate relationships between tissue metal burden and circulating biomarkers of oxidative DNA damage and global DNA methylation in peripheral blood.

**Methods:**

Fifty adult patients with newly diagnosed primary colon cancer undergoing surgical resection were included. Concentrations of lead (Pb), cadmium (Cd), mercury (Hg), arsenic (As), chromium (Cr), and titanium (Ti) were measured in paired colon tissue samples using ICP-MS. Global DNA methylation was assessed in colon tissue and peripheral blood using ELISA-based quantification of 5-methylcytosine, while oxidative DNA damage in peripheral blood lymphocytes was measured using the comet assay (tail moment). Paired comparisons were conducted using the Wilcoxon signed-rank test. Multivariable linear regression models adjusted for age, sex, body mass index, education, and tumor location were used to evaluate associations between metals, a composite Metal Burden Index, DNA methylation, and oxidative DNA damage.

**Results:**

Cadmium concentrations were significantly lower in cancerous compared with non-cancerous colon tissue (median 0.011 vs. 0.019 μg/g wet weight; *p* = 0.005), while no significant differences were observed for Pb, Hg, As, Cr, or Ti. No statistically significant associations were found between individual tissue metals or the Metal Burden Index and global DNA methylation in either colon tissue type. Tissue metal concentrations were generally not associated with blood global DNA methylation. An inverse association was observed between Hg concentration in cancerous tissue and blood comet assay tail moment (β = −0.582; 95% CI: −1.004 to −0.160; *p* = 0.008), which should be interpreted cautiously. Correlations between tissue and blood-based epigenetic and genotoxic biomarkers were weak.

**Conclusions:**

In this cross-sectional study, metal accumulation in colon tissue was not consistently associated with global DNA methylation in tissue or blood. The limited concordance between tissue-based metal concentrations and circulating epigenetic or genotoxic biomarkers highlights the complexity of exposure–biomarker relationships in established colon cancer and suggests that blood-based markers may inadequately reflect localized tissue-level processes, with implications for environmental exposure assessment and public health biomonitoring.

## Introduction

Colorectal cancer (CRC) remains a major global health burden and is among the most common malignancies worldwide (1). In Saudi Arabia, CRC is among the most common cancers, and patients have been reported to present at a younger age compared with Western populations (2, 3). While inherited susceptibility contributes to CRC risk, geographic variation in incidence and temporal trends underscore the importance of environmental and lifestyle-related determinants, including dietary exposures (4, 5). Colon cancer represents the majority of CRC cases and differs from rectal cancer in both biology and clinical management, particularly with respect to the routine use of neoadjuvant chemotherapy and radiotherapy in rectal tumors (6).

Diet-related cancer risk may reflect both dietary patterns and exposure to environmental pollutants (7). Toxic and trace metals such as lead (Pb), cadmium (Cd), mercury (Hg), arsenic (As), and chromium (Cr) are of particular concern because of their presence as food contaminants and their established toxicological profiles (8). The Agency for Toxic Substances and Disease Registry (ATSDR) has ranked several heavy metals—including inorganic As (1), Pb (2), Hg (3), Cd (7), and hexavalent Cr (17)—among priority hazardous substances based on their frequency at contaminated sites, toxicity, and potential for human exposure (9). These metals have been widely investigated in environmental epidemiology and human biomonitoring studies due to their persistence, bioaccumulation potential, and documented ability to induce molecular alterations relevant to carcinogenesis (10, 11). Experimental and mechanistic studies suggest that metal exposure may contribute to carcinogenesis through several pathways, including oxidative stress generation, induction of DNA damage, disruption of DNA repair mechanisms, and epigenetic alterations affecting gene regulation (12). Consistent with these mechanisms, several heavy metals have been shown to induce cytotoxic and genotoxic effects in human cell lines relevant to gastrointestinal tissues (13). In human studies, differences in metal concentrations between cancerous and non-cancerous colorectal tissues have been reported, but findings remain inconsistent across investigations (14, 15), likely reflecting heterogeneity in exposure levels, disease characteristics, and analytical approaches. Titanium dioxide (TiO_2_; E171), widely used as a whitening agent in food and consumer products, has also been implicated in colon tumor promotion and preneoplastic changes in experimental models (16–19). Population-level ecological studies have further reported associations between environmental metal exposure and colon cancer incidence, supporting the potential relevance of dietary and environmental metal exposure in colorectal carcinogenesis (20).

Mechanistically, metal-induced carcinogenesis is strongly linked to oxidative stress, where excess reactive oxygen species (ROS) can damage lipids, proteins, and DNA and promote genetic instability (21–24). Genotoxicity refers to adverse effects on the genome of living cells that may result in mutations, chromosomal alterations, or other DNA damage events that contribute to carcinogenesis (25). Oxidative DNA damage has been associated with CRC risk and progression in epidemiologic studies, including evidence linking oxidative DNA/RNA damage biomarkers to CRC incidence and severity (26, 27). The alkaline single-cell gel electrophoresis (comet) assay provides a sensitive measure of DNA strand breaks in individual cells and is widely used for assessing genotoxicity in human biomonitoring and exposure assessment studies (28).

Beyond direct genotoxic effects, metals may contribute to carcinogenesis through epigenetic dysregulation, particularly through changes in DNA methylation. Aberrant DNA methylation is a hallmark of colorectal carcinogenesis, accumulating across the adenoma–carcinoma sequence and contributing to altered gene regulation and tumor behavior (29, 30). Global and gene-specific methylation alterations have been explored as biomarkers for CRC detection and prognosis, including the potential utility of blood-based methylation signatures as minimally invasive markers (31, 32). Lifestyle factors such as smoking and alcohol use have also been linked to CRC-associated methylation patterns, supporting the sensitivity of methylation to exposure-related influences (33–36).

Despite experimental evidence linking metal exposure to oxidative stress and epigenetic dysregulation, human data integrating tissue-level metal accumulation with both local, and circulating genotoxic and epigenetic biomarkers remain limited. In colorectal cancer in particular, it is unclear whether metal concentrations within the tumor microenvironment are reflected by circulating biomarkers of oxidative DNA damage and global DNA methylation, or whether tumor-specific metabolic and epigenetic alterations attenuate the relationship between tissue exposure and systemic measures, as has been observed for other circulating epigenetic biomarkers in colorectal cancer (37). Clarifying this relationship is important for interpretating circulating biomarkers used in environmental exposure assessment and public health surveillance.

Therefore, this study aimed to (i) characterize concentrations of selected toxic and trace metals in paired cancerous and adjacent non-cancerous colon tissues; (ii) evaluate associations between tissue metal concentrations and global DNA methylation within colon tissue; and (iii) examine relationships between tissue metal burden and systemic biomarkers of oxidative DNA damage and global DNA methylation measured in peripheral blood. By integrating tissue-based exposure assessment with circulating genotoxic and epigenetic endpoints, this study seeks to improve understanding of exposure–biomarker relationships in the context of established colon cancer and to inform the interpretation of commonly used biomarkers in environmental health research.

## Materials and methods

### Study design and participants

This study included 50 Saudi adult patients (≥18 years) with newly diagnosed, histologically confirmed primary colon cancer who underwent surgical resection at King Faisal Specialist Hospital & Research Center. Patients with rectal cancer were excluded, as neoadjuvant chemotherapy and/or radiotherapy are routinely administered before surgery for rectal tumors and could confound the assessment of metal accumulation, oxidative DNA damage, and DNA methylation. Additional exclusion criteria included prior chemotherapy or radiotherapy before surgery, metastatic disease, and pregnancy. Tumor location within the colon was abstracted from surgical and pathology reports and categorized anatomically as proximal (right-sided) or distal (left-sided, sigmoid, and rectosigmoid) colon for analysis.

All participants provided written informed consent prior to enrollment. The study protocol was approved by the institutional Research Ethics Committee (RAC # 2200005), and confidentiality was maintained through the use of unique study identifiers.

### Tissue and blood sample collection

Paired colon tissue specimens were collected from each participant at the time of surgical resection. Cancerous tissue samples were obtained from the tumor mass, while non-cancerous tissue samples were obtained from macroscopically normal colon tissue located at least 10 cm from the tumor margin. Tissue samples were immediately snap-frozen in liquid nitrogen and stored at −80 °C until analysis.

Peripheral venous blood samples were collected in EDTA tubes prior to surgery for DNA extraction, assessment of oxidative DNA damage, and global DNA methylation analysis.

### Metal analysis in colon tissues

Colon tissue samples were prioritized for trace metal analysis. Concentrations of Pb, Cd, Hg, As, Cr, and titanium (Ti) were measured in acid-digested colon tissue samples using inductively coupled plasma–mass spectrometry (ICP-MS; NexION^®^ 2000, PerkinElmer). Quality assurance and quality control procedures included the use of internal standards, multi-element calibration standards prepared from high-purity stock solutions, matrix-matched spike-recovery experiments, and within-run and between-run precision assessments. A certified reference material was not used because a suitable, non-expired, matrix-matched certified reference material matching the colon tissue matrix was not available at the time of analysis. Instead, method performance was evaluated using spiked sheep colon tissue digests to assess analytical accuracy and precision under matrix conditions comparable to those of the study samples; these evaluations demonstrated acceptable analytical recovery and reproducibility across all measured metals.

Approximately 0.05 g of frozen colon tissue was digested with 3 mL of concentrated nitric acid (HNO_3_; Fisher Scientific, PA, USA) in acid-cleaned, labeled Teflon digestion vessels. Tissue samples were analyzed on a wet-weight basis, as frozen tissue was digested directly without prior drying, and a standardized tissue mass of approximately 0.05 g was used for digestion. Samples were incubated overnight at 85 °C, cooled to room temperature, and subsequently treated with 1 ml hydrogen peroxide, followed by an additional digestion step at 85 °C for 1 hour. After cooling, the digests were transferred to 15 ml polypropylene tubes and diluted to a final volume of 10 ml with deionized water.

Multi-element calibration standards were prepared from a commercial multi-element standard solution (100 μg/ml in 5% HNO_3_) and a Hg standard solution (10 μg/ml). Working calibration standards covering the analytical range were prepared in 2% HNO_3_. Gold was added to the acidic diluent and wash solutions to stabilize Hg and minimize memory effects during analysis. An acidic diluent containing HNO_3_, Triton X-100 (Sigma-Aldrich^™^, MO, USA), methanol (Fisher Scientific, PA, USA; all v/v), and gold was used for final sample preparation before ICP-MS analysis.

An aliquot of 50 μL of each tissue digest was diluted with acidic diluent consisting of 0.5% nitric acid, 0.05% Triton X-100, and 2% methanol (all v/v). Internal standards were added to all samples to correct for instrumental drift and matrix effects: iridium (^193^Ir) for Pb, Cd, Hg, and As, and rhodium (^103^Rh) for Cr and Ti. Metal quantification was performed by ICP-MS, and diluent signal intensities were subtracted from calibrators, quality control samples, and patient specimens.

Calibration curves ranged from 0.25 to 4 μg/L for Pb, Cd, Hg, and As, and from 0.5 to 8 μg/L for Cr and Ti. All calibration curves demonstrated excellent linearity, with correlation coefficients (r^2^) ≥0.999. Analytical performance was further evaluated through spike-recovery experiments and replicate precision analyses. Between-run precision was assessed by digesting sheep colon tissue and spiking the digests with three concentration levels (0.75, 1.5, and 3 μg/L for Pb, Cd, Hg, and As; and 1.5, 3, and 6 μg/L for Cr and Ti), which were analyzed alongside patient samples. Spike recovery ranged from 98 to 99% with relative standard deviations (%RSD) of 5–7% for Pb, Cd, Hg, and As, and 9%8–101% with %RSD of 2–4% for Cr and Ti.

Within-run precision was evaluated by performing 10 replicate analyses of spiked sheep colon tissue digests. The %RSD values ranged from 2%−7% for Pb, Cd, Hg, and As, and from 3%−5% for Cr and Ti. Method detection limits (MDLs) in the analytical solution (μg/L) were 0.017 for Pb, 0.0005 for Cd, 0.073 for Hg, 0.002 for As, 0.131 for Cr, and 0.039 for Ti. Based on the digestion procedure used (0.05 g tissue diluted to a final volume of 10 mL), the corresponding MDLs in tissue units (μg/g) were 0.0034 for Pb, 0.0001 for Cd, 0.0146 for Hg, 0.0004 for As, 0.0262 for Cr, and 0.0078 for Ti.

### Assessment of oxidative DNA damage in peripheral blood

Systemic oxidative DNA damage was assessed in peripheral blood lymphocytes using the alkaline single-cell gel electrophoresis (comet) assay, following the established protocol of Singh, McCoy, Tice, and Schneider (28). Peripheral blood lymphocytes were isolated from whole blood by collection of the buffy coat fraction following centrifugation. The isolated cells were cryopreserved in a commercially available freezing medium (Sigma-Aldrich, MO, USA) containing 10% DMSO as a cryoprotectant and stored at −80 °C until analysis. For comet assay analysis, samples were rapidly thawed in a 37 °C water bath and processed immediately, consistent with established protocols for cryopreserved blood preparations (38). After thawing, the isolated lymphocytes were embedded in agarose for comet assay evaluation. Comet images were captured using a fluorescence microscope equipped with appropriate excitation and barrier filters. For each sample, 25 randomly selected, intact, non-overlapping nucleoids were analyzed using Comet Assay IV image analysis software with a monochrome CCD IEEE1394 FireWire video camera (Perceptive Instruments, Halstead, UK) (39). Cells were selected from different areas of the gel to minimize selection bias and ensure representative sampling. Image analysis was performed at 20 × magnification using a fluorescence optical microscope (Eclipse Ti-E; Nikon, Japan) equipped with excitation (465 nm) and barrier (595 nm) filters. Five parameters were measured, including head length, tail length, head intensity, % tail DNA, and tail moment. Tail moment was selected as the primary measure of DNA damage, because it integrates both the extent of DNA migration and the proportion of DNA present in the comet tail and is widely used as a sensitive parameter for assessing DNA strand breaks in human biomonitoring studies (40). Positive and negative internal controls were not included because suitable control materials were not consistently available in usable condition at the time of analysis; this is acknowledged as a limitation of the assay. In addition, although 25 nucleoids per sample were analyzed, all samples were processed and scored under the same experimental conditions using a standardized protocol, and only intact, randomly selected, non-overlapping nucleoids were included in the analysis to reduce scoring bias.

### Global DNA methylation analysis

Genomic DNA was extracted from paired colon cancer and non-cancerous tissue biopsies and from peripheral blood samples. DNA from colon tissues was isolated using a proteinase K digestion followed by phenol–chloroform extraction and ethanol precipitation. Briefly, tissue samples were digested overnight at 55 °C in a lysis buffer containing Tris-HCl, EDTA, NaCl, SDS, and proteinase K, followed by phenol–chloroform–isoamyl alcohol extraction, ethanol precipitation, washing with 70% ethanol, and resuspension in TE buffer. DNA from peripheral blood samples was extracted using the QIAamp Blood Midi Kit (Qiagen, Germany) according to the manufacturer's spin-column protocol. DNA concentration and purity for all samples were assessed using a NanoDrop spectrophotometer; however, DNA integrity was not evaluated separately using gel electrophoresis or an equivalent method, as integrity represents a distinct quality parameter from spectrophotometric purity (41).

Due to variability in residual tissue availability after trace metal analysis, global DNA methylation measurements were successfully obtained in colon tissue from 41 participants, yielding 82 paired cancerous and non-cancerous tissue samples. Global DNA methylation in peripheral blood was successfully analyzed in 42 participants. Global DNA methylation was assessed in colon tissue to reflect local epigenetic alterations and in peripheral blood to provide a systemic biomarker for comparison with tissue-based measures.

Global DNA methylation was quantified using a competitive enzyme-linked immunosorbent assay (ELISA) based on the detection of 5-methyl-2′-deoxycytidine (5-mC) (Global DNA Methylation ELISA Kit; Cell Biolabs, USA), following the manufacturer's instructions. Briefly, purified genomic DNA was denatured at 95 °C for 5 min and rapidly cooled on ice. Denatured DNA was enzymatically digested to single nucleotides using nuclease P1, followed by dephosphorylation with alkaline phosphatase. The resulting nucleotide mixtures were added to microplate wells pre-coated with a 5-methylcytosine DNA conjugate and incubated with a monoclonal anti-5-methylcytosine antibody. After incubation with a horseradish peroxidase–conjugated secondary antibody, color development was achieved using the supplied substrate, and absorbance was measured at 450 nm.

A standard curve generated from supplied 5-methylcytosine standards was used to calculate global DNA methylation levels, which were expressed as relative methylation values.

### Statistical analysis

Descriptive analyses were conducted using an available-case approach. Metal concentrations exhibited skewed distributions and are therefore summarized as medians and interquartile ranges (IQRs). Values below the MDL were treated as non-detects and replaced with one-half of the corresponding MDL prior to statistical analysis. Paired comparisons between cancerous and non-cancerous colon tissues were performed using the Wilcoxon signed-rank test.

Multivariable regression models were fitted using complete-case observations for the specified outcome and covariates. To minimize the risk of model overfitting given the sample size, covariates included in regression models were selected based on biological relevance and prior literature rather than purely data-driven approaches (42). Adjusted models were therefore restricted to key potential confounders to preserve model stability.

Associations between tissue metal concentrations and global DNA methylation in colon tissue were evaluated using multivariable linear regression models, fitted separately for non-cancerous and cancerous tissues. Results are presented as unstandardized regression coefficients (β) with corresponding 95% confidence intervals (95% CI) and *p*-values. Metal concentrations were log-transformed prior to inclusion in regression models to reduce right skewness. Models were adjusted for age, sex, body mass index (BMI), educational attainment, and tumor location, with distal colon specified as the reference category for tumor location. Tumor location was modeled using dummy variables, with distal colon specified as the reference category and indicator variables created for proximal colon and total colon involvement.

An exploratory Metal Burden Index was constructed as the mean of standardized (z-scored) log-transformed concentrations of Pb, Cd, Hg, As, Cr, and Ti and was evaluated using multivariable linear regression with the same covariate adjustment.

Peripheral blood biomarkers, including global DNA methylation and oxidative DNA damage assessed by comet assay tail moment, were analyzed at the participant level. Tail moment values were log-transformed before analysis. Associations between tissue metal concentrations and blood biomarkers were examined using multivariable linear regression models, fitted separately for metals measured in non-cancerous and cancerous tissues and adjusted for the same set of covariates.

Spearman correlation analyses were conducted to examine the concordance between tissue-based and blood-based biomarkers, including relationships between tissue DNA methylation and blood-based epigenetic and genotoxic measures.

All statistical analyses were performed using IBM SPSS Statistics for Windows, Version 23.0 (IBM Corp., Armonk, NY, USA), and a two-sided *p*-value <0.05 was considered statistically significant. Given the exploratory nature of the analyses, *p*-values were interpreted cautiously and no formal correction for multiple comparisons was applied.

## Results

### Participant characteristics

A total of 50 patients with primary colon cancer were included in the study. The mean age of participants was 56.9 ± 11.8 years, and 52% were male. The mean BMI was 28.7 ± 5.0 kg/m^2^. Most participants were married (78%) and resided in the central region of Saudi Arabia (44%). Tumors were most commonly located in the distal colon (60%), followed by the proximal colon (30%), with 10% involving the total colon. The majority of tumors were low-to-intermediate grade (grades 1–2, 88%), and lymphovascular invasion was present in 42% of cases. Due to variability in tissue availability after metal analysis and DNA extraction, global DNA methylation and peripheral blood biomarker analyses were conducted in subsets of participants, as specified in subsequent sections. Additional demographic, lifestyle, and clinicopathologic characteristics are summarized in Table 1.

**Table 1:** Participant characteristics (*n* = 50).

Characteristic	Mean	*n* (%)
Age (years)	56.9 ± 11.8	
BMI (kg/m^2^)	28.7 ± 5.0	
Duration of living in the region (years)	51.3 ± 16.9	
Sex (male/female)		26 (52%)/24 (48%)
Marital status (married/single/divorced/widowed)		39 (78%)/3 (6%)/2 (4%)/6 (12%)
Living region (central/northern/southern/eastern/western)		22 (44%)/8 (16%)/4 (8%)/8 (16%)/9 (18%)
Education attainment (≤ 12 years/>12 years)		21 (42%)/29 (58%)
Work status (current/past/never)		12 (24%)/17 (34%)/21 (42%)
Total family income in Saudi riyals (SAR) (<5,000/5,000-10,000/> 10,000/refused or unknown)		8 (16%)/11 (22%)/22 (44%)/9 (18%)
Herbal remedies (yes/no)		8 (16%)/42 (84%)
Smoking status (current/former/never)		7 (14%)/2 (4%)/41 (84%)
Seafoods consumption (yes/no)		42 (84%)/8 (16%)
Red meats consumption (yes/no)		47 (94%)/3 (6%)
Smoked food consumption (yes/no)		12 (24%)/38 (76%)
Use of incense (yes/no)		46 (92%)/4 (8%)
Tumor location (proximal/distal/total colon)		15 (30%)/30 (60%)/5 (10%)
Tumor grade (1–2/3)		44 (88%)/6 (12%)
Depth of invasion (pT1/pT2–4)		1 (2%)/49 (98%)
Lympho-vascular invasion (present/absent)		21 (42%)/29 (58%)
Perineural invasion (present/absent)		13 (26%)/37 (74%)
Lymph node invasion (present/absent)		23 (46%)/27 (54%)
Family cancer (yes/no)		22 (44%)/28 (56%)

Percentages may not sum to 100 due to rounding.

### Metal concentrations in paired colon tissues

Concentrations of Pb, Cd, Hg, As, Cr, and Ti were measured in paired non-cancerous and cancerous colon tissue samples from all 50 participants. Detection frequencies of the analyzed metals and method detection limits are presented in Supplementary Table S1. When comparing paired tissues, Cd concentrations were significantly lower in cancerous tissue compared with non-cancerous tissue (median 0.011 vs. 0.019 μg/g wet weight; *p* = 0.005). No statistically significant differences were observed between cancerous and non-cancerous tissues for Pb, Hg, As, Cr, or Ti (all *p* > 0.05). Detailed metal concentrations and paired comparison results are presented in Table 2.

**Table 2:** Metal concentrations (μg/g wet weight) in paired non-cancerous and cancerous colon tissues (*n* = 50).

Metal	Non-cancerous tissue		Cancerous tissue		*p*-value^††^
	Median	IQR^†^	Median	IQR^†^	
Pb	0.139	0.504	0.126	0.333	0.631
Cd	0.019	0.051	0.011	0.059	0.005
Hg	0.007	0.000^‡^	0.007	0.000^‡^	0.463
As	0.0003	0.092	0.0003	0.111	0.715
Cr	1.324	2.044	1.728	2.102	0.534
Ti	0.878	0.920	0.973	1.434	0.286

^†^IQR: interquartile range, defined as the 25th and 75th percentiles.

^††^*p*-values obtained using the Wilcoxon signed-rank test for paired samples.

^‡^The interquartile range of 0.000 reflects that most Hg concentrations were below the method detection limit (MDL). Values below the MDL were substituted with one-half of the corresponding MDL (MDL/2) prior to statistical analysis. For Hg, the analytical-solution MDL was 0.073 μg/L, corresponding to 0.0146 μg/g tissue after conversion based on the digestion of 0.05 g tissue to a final volume of 10 ml; thus, the substituted value for non-detects was 0.0073 μg/g, which appears as 0.007 after rounding.

### Tissue metals and global DNA methylation in colon tissue

Global DNA methylation measurements in colon tissue were available for 41 participants. Multivariable linear regression models were fitted separately for non-cancerous and cancerous tissues to evaluate associations between tissue metal concentrations and global DNA methylation, adjusting for age, sex, BMI, education, and tumor location.

No statistically significant associations were observed between individual tissue metal concentrations and global DNA methylation in either non-cancerous or cancerous colon tissue. Effect estimates were generally small, and 95% confidence intervals included the null value for all metals examined. Similarly, an exploratory composite Metal Burden Index, calculated as the mean of standardized log-transformed metal concentrations, was not significantly associated with tissue DNA methylation in either tissue type. These results are shown in Table 3.

**Table 3:** Associations between tissue metals and tissue DNA methylation (adjusted models, *n* = 41).

Metal	Non-cancerous tissue				Cancerous tissue			
	β	95% CI (L)	95% CI (U)	*p*	β	95% CI (L)	95% CI (U)	*p*
Pb	0.091	−0.441	0.623	0.730	−0.035	−1.201	1.132	0.952
Cd	−0.006	−0.675	0.663	0.986	0.581	−0.262	1.424	0.170
Hg	−0.950	−3.080	1.181	0.371	−0.159	−5.504	5.186	0.952
As	−0.010	−0.508	0.489	0.969	−0.170	−0.969	0.629	0.668
Cr	−0.252	−1.251	0.747	0.611	−1.101	−2.846	0.644	0.208
Ti	0.176	−0.850	1.201	0.730	0.237	−1.166	1.641	0.733
Metal Burden Index	−0.430	−3.602	2.743	0.785	0.050	−5.790	6.394	0.920

Models adjusted for age, sex, BMI, educational attainment, and tumor location. Metal concentrations were log-transformed prior to analysis. β represents the unstandardized regression coefficient for log-transformed metal concentrations.

### Tissue metals and global DNA methylation in peripheral blood

Associations between tissue metal concentrations and global DNA methylation in peripheral blood were evaluated among the same 41 participants using multivariable linear regression models with identical covariate adjustment, reflecting complete-case observations for both tissue metal concentrations and blood methylation outcomes.

Overall, no consistent associations were observed between tissue metal concentrations and blood global DNA methylation. For most metals, effect estimates were close to zero, and confidence intervals spanned the null. An inverse association was observed between Cd concentration in cancerous tissue and blood DNA methylation (β = −0.113; 95% CI: −0.225, −0.001; *p* = 0.047), while no corresponding association was seen for Cd in non-cancerous tissue. The composite Metal Burden Index was not associated with blood DNA methylation in either tissue category. Results are summarized in Table 4.

**Table 4:** Associations between tissue metals and blood DNA methylation (adjusted models, *n* = 41).

Metal	Non-cancerous tissue				Cancerous tissue			
	β	95% CI (L)	95% CI (U)	*p*	β	95% CI (L)	95% CI (U)	*p*
Pb	0.007	−0.098	0.113	0.887	−0.030	−0.162	0.102	0.644
Cd	−0.098	−0.226	0.03	0.127	−0.113	−0.225	−0.001	**0.047**
Hg	0.042	−0.386	0.47	0.843	0.111	−0.386	0.609	0.652
As	0.072	−0.031	0.175	0.164	0.053	−0.042	0.148	0.264
Cr	0.008	−0.241	0.257	0.948	−0.024	−0.231	0.182	0.812
Ti	−0.046	−0.26	0.168	0.663	−0.019	−0.176	0.139	0.812
Metal Burden Index	−0.004	−0.641	0.633	0.99	−0.147	−0.753	0.458	0.624

Models adjusted for age, sex, BMI, educational attainment, and tumor location. Metal concentrations were log-transformed prior to analysis. β represents the unstandardized regression coefficient.

Bold values indicate statistically significant associations (*p* < 0.05).

### Tissue metals and oxidative DNA damage in peripheral blood

Systemic oxidative DNA damage was assessed using the comet assay tail moment in peripheral blood lymphocytes. Tail moment values were log-transformed before analysis. Multivariable regression models were fitted to examine associations between tissue metal concentrations and blood tail moment. Analyses were restricted to participants with complete data on tissue metal concentrations, comet assay parameters, and covariates (*n* = 41).

For metals measured in non-cancerous tissue, no statistically significant associations with blood tail moment were observed. In contrast, higher Hg concentrations in cancerous tissue were significantly associated with lower blood tail moment (β = −0.582; 95% CI: −1.004 to −0.160; *p* = 0.008). No other metals, nor the Metal Burden Index, showed significant associations with blood tail moment. These findings are presented in Table 5.

**Table 5:** Associations between tissue metals and blood DNA damage—tail moment (adjusted models, *n* = 41).

Metal	Non-cancerous tissue				Cancerous tissue			
	β	95% CI (L)	95% CI (U)	*p*	β	95% CI (L)	95% CI (U)	*p*
Pb	−0.006	−0.105	0.092	0.897	0.004	−0.120	0.128	0.954
Cd	0.022	−0.102	0.146	0.725	−0.001	−0.106	0.104	0.983
Hg	−0.266	−0.657	0.125	0.176	−0.582	−1.004	−0.160	**0.008**
As	0.018	−0.079	0.114	0.712	−0.001	−0.092	0.089	0.975
Cr	−0.003	−0.234	0.228	0.979	0.077	−0.114	0.268	0.418
Ti	−0.012	−0.212	0.189	0.905	−0.025	−0.172	0.121	0.73
Metal Burden Index	−0.103	−0.69	0.483	0.723	−0.181	−0.748	0.386	0.521

Models adjusted for age, sex, BMI, educational attainment, and tumor location. Metal concentrations and tail moment were log-transformed prior to analysis. β represents the unstandardized regression coefficient.

Bold values indicate statistically significant associations (*p* < 0.05).

In supplementary analyses using percent tail DNA (%DNA) as an alternative comet assay parameter, results were generally consistent with those observed for tail moment, with a similar inverse association observed for Hg in cancerous tissue. No additional statistically significant associations were identified (Supplementary Table S2).

### Relationships between blood DNA methylation, tissue DNA methylation, and oxidative DNA damage

In cancerous tissue samples, Spearman correlation analyses were conducted to examine relationships between global DNA methylation in peripheral blood, oxidative DNA damage measured by blood comet assay tail moment, and global DNA methylation in colon tissue.

Blood global DNA methylation was not significantly correlated with blood comet assay tail moment (ρ = 0.056, *p* = 0.729; *n* = 41). A moderate inverse correlation was observed between blood global DNA methylation and DNA methylation measured in cancerous colon tissue; however, this association did not reach statistical significance (ρ = −0.299, *p* = 0.096; *n* = 32).

No significant association was observed between blood comet assay tail moment and DNA methylation in cancerous colon tissue (ρ = −0.016, *p* = 0.929; *n* = 33).

Overall, these findings indicate limited correspondence between blood-based systemic biomarkers and epigenetic alterations in cancerous colon tissue.

## Discussion

In this study, we assessed concentrations of selected metals in paired non-cancerous and cancerous colon tissues and examined their relationships with global DNA methylation in tissue and peripheral blood, as well as systemic oxidative DNA damage assessed by the comet assay. By integrating tissue-level exposure measurements with circulating epigenetic and genotoxic biomarkers, this study aimed to evaluate whether localized metal accumulation within the colon is reflected in commonly used circulating biomarkers and whether such exposure is associated with global epigenetic alterations. Overall, Cd concentrations were lower in cancerous compared with paired non-cancerous colon tissue, whereas other metals showed no consistent tissue-level differences. Beyond this tissue contrast, metal concentrations were generally not associated with global DNA methylation in either colon tissue or peripheral blood, and overall concordance between tissue- and blood-based biomarkers was limited. An inverse association between Hg concentration in cancerous tissue and blood comet assay tail moment was observed in multivariable analysis; however, given its isolated nature and the absence of consistent supporting associations, this finding should be interpreted with caution.

From a mechanistic toxicology perspective, the limited associations observed between tissue metal concentrations and global DNA methylation or systemic oxidative DNA damage likely reflect the biological and metabolic complexity of established malignancy rather than an absence of metal-related effects. Toxic and trace metals have been shown to contribute to carcinogenesis through oxidative stress, interference with DNA repair pathways, and epigenetic dysregulation (23, 24, 29). However, following malignant transformation, tumor cells undergo extensive metabolic, redox, and epigenetic reprogramming, which may alter metal uptake, sequestration, and biological responses independently of external exposure (43). Consequently, tissue metal accumulation may not be directly reflected in circulating biomarkers, particularly when global epigenetic endpoints are assessed, underscoring the limitations of systemic markers for capturing localized effects within the tumor microenvironment.

### Metal concentrations in paired colon tissues

Among the metals analyzed, Cd concentrations were significantly lower in cancerous tissue compared with paired non-cancerous tissue, while no significant differences were observed for Pb, Hg, As, Cr, or Ti. Altered trace element profiles between malignant and adjacent colorectal tissues have been reported previously, although inconsistencies across elements and studies are common (44, 45). In contrast, Sohrabi et al. reported higher concentrations of several trace elements, including Pb, and Cr, in cancerous compared with non-cancerous colorectal tissues, highlighting substantial variability related to study populations, tumor characteristics, exposure sources, and analytical methodologies (14). Differences in Cd concentrations may reflect tumor-related alterations in cellular metabolism, metal transport, or sequestration rather than differences in external exposure. Experimental and clinical evidence indicates that malignant transformation can disrupt intracellular metal homeostasis, while tumor heterogeneity and microenvironment variability may contribute to heterogeneous metal accumulation patterns within tumor tissue (46, 47). In addition, intracellular Cd distribution may be influenced by metallothioneins, cysteine-rich metal-binding proteins that regulate metal homeostasis and detoxification. Metallothioneins have been reported to be upregulated in several malignancies, including colorectal cancer, and may alter intracellular sequestration and redistribution of Cd within tumor cells (21, 48). Such alterations in metal-binding and storage mechanisms could contribute to lower measurable Cd concentrations in tumor tissue despite continued environmental exposure.

Furthermore, differences in tissue composition between tumor tissue and adjacent mucosa—including variations in cellular density, stromal content, and water content—may influence measured metal concentrations when expressed on a wet-weight basis. In the present study, metal concentrations were expressed on a wet-weight basis, an approach commonly used in biomonitoring studies of trace elements in human tissues to preserve the physiological state of fresh samples and facilitate comparison across biological matrices (49). However, normalization to dry weight or protein content was not performed because additional measurements were not feasible after tissue digestion for metal analysis and DNA extraction.

Previous studies have shown that malignant tissues may exhibit substantially different wet-to-dry ratios compared with non-tumorous tissues, which can affect the apparent concentration of trace elements measured in fresh tissue samples (50). Because malignant tissues often exhibit altered structural and biochemical characteristics compared with non-tumorous mucosa (51), normalization approaches based on dry weight or protein content could potentially influence the interpretation of tissue metal differences. In addition, recent analyses have highlighted that discrepancies in reported tissue metal concentrations across studies may partly arise from differences in pre-analytical and analytical methodologies used for tissue preparation and metal quantification (52).

Another possible explanation is that histologically normal mucosa adjacent to tumors may already harbor molecular alterations associated with field cancerization (53, 54). In colorectal carcinogenesis, genetic and epigenetic changes may extend beyond the visible tumor margins, meaning that adjacent tissue may not represent a completely unaffected reference for comparison. Such field effects could potentially influence local metal distribution patterns between tumor and surrounding mucosa.

Future studies incorporating such normalization strategies may help clarify whether observed differences reflect biological redistribution of metals or variations in tissue composition.

### Tissue metals and global DNA methylation in colon tissue

Although metals have been linked to epigenetic dysregulation in experimental and epidemiologic studies, we observed no statistically significant associations between individual tissue metal concentrations and global DNA methylation in either non-cancerous or cancerous colon tissue. These findings are consistent with prior human studies reporting weak or absent associations between metal exposure and global methylation measures (55), despite growing evidence that metals may preferentially induce locus-specific rather than global methylation changes (56).

Global DNA methylation represents a relatively coarse epigenetic endpoint and may not capture subtle, gene-specific methylation alterations that are more directly relevant to carcinogenesis, which often involves localized hypermethylation at promoters, enhancers, or CpG islands rather than uniform genome-wide changes (57). Moreover, epigenetic alterations associated with metal exposure may occur early in the carcinogenic process, including during premalignant stages, whereas epigenetic patterns measured in established tumors may reflect more stable, clonally selected states that are less indicative of antecedent exposure-related changes (58). This temporal and biological disconnect may partly explain the absence of detectable associations in cross-sectional analyses of tumor tissue. Limited statistical power due to the modest sample size may have further constrained the ability to detect small effect sizes.

### Tissue metals and blood-based epigenetic and genotoxic biomarkers

Overall, tissue metal concentrations were not consistently associated with blood global DNA methylation or comet assay parameters. An inverse association between Cd concentration in cancerous tissue and blood DNA methylation was observed in the adjusted models; however, this association reached borderline statistical significance (*p* = 0.047), with a confidence interval close to the null value, and was identified in the context of multiple exploratory analyses without formal correction for multiple comparisons. Moreover, this association was observed only for blood DNA methylation and not for DNA methylation measured in colon tissue, suggesting that the finding may reflect systemic variability rather than a consistent tissue-level epigenetic effect. Accordingly, this result should be interpreted cautiously and considered hypothesis-generating, requiring confirmation in larger studies examining cadmium-related epigenetic alterations (56).

Although blood-based biomarkers are widely used in environmental epidemiology, their capacity to reflect tissue-specific exposure or localized biological effects is inherently limited, as they primarily capture systemic rather than organ-specific responses (59). This distinction is particularly relevant in the present study, where metals were measured in colon tissue while epigenetic and genotoxic biomarkers were assessed in peripheral blood. One possible pathway is that metal accumulation in colon tissue may induce localized oxidative stress or epigenetic alterations that subsequently influence systemic inflammatory or oxidative signaling, which may be reflected in circulating biomarkers (24, 58). These findings underscore the importance of exposure compartmentalization, whereby tissue-level accumulation and biological effects may not be adequately captured by peripheral biomarkers.

The comet assay is nevertheless a sensitive and well-established indicator of oxidative DNA damage in human biomonitoring studies, and associations between metal exposure and DNA strand breaks have been reported across diverse populations (60, 61). Telomeres protect chromosomal integrity and maintain genomic stability, and alterations in telomere length have been associated with oxidative stress, environmental exposures, and carcinogenic processes. Experimental studies have shown that exposure to metal-containing particles can modify telomere length and epigenetic regulation, suggesting that telomere dynamics may represent an additional biomarker of metal-induced genotoxicity and disease risk. Future studies integrating tissue metal measurements with telomere length assessment may therefore provide further insight into mechanisms linking metal exposure with colorectal carcinogenesis (62–64).

In the present study, an inverse association between Hg concentration in cancerous tissue and blood comet assay tail moment was observed. While Hg has demonstrated genotoxic potential in experimental systems (65), this isolated finding should be interpreted with particular caution given the modest sample size and the high proportion of Hg concentrations below the MDL, which may have limited the stability and precision of Hg-specific estimates. One possible explanation is that adaptive redox and antioxidant responses within established tumors may modulate measurable systemic oxidative DNA damage, resulting in lower comet assay indices despite ongoing exposure (48). The compartment-specific nature of tissue metal accumulation and systemic biomarker responses, together with the possibility of chance findings, limits definitive mechanistic interpretation.

### Relationships between tissue and blood epigenetic markers

Exploratory analyses revealed limited correspondence between DNA methylation measured in colon tissue and peripheral blood. Blood-based DNA methylation is commonly used as a surrogate for target tissues; however, substantial tissue specificity in methylation patterns has been consistently documented, and concordance between blood and other organs is often modest (66–68). The absence of strong correlations observed in this study reinforces the tissue-specific nature of epigenetic regulation and highlights the need for caution when interpreting blood-based epigenetic biomarkers in relation to tissue-level biological processes, particularly in colorectal cancer (69).

### Strengths and limitations

Key strengths of this study include the use of paired colon tissue samples, which minimizes inter-individual variability, and the integration of tissue-specific exposure assessment with systemic epigenetic and genotoxic biomarkers. The application of conservative statistical methods and exploratory mixture analyses further enhances the robustness and interpretability of the findings.

Limitations of this study include the modest sample size, the use of global rather than locus-specific DNA methylation measures, and the cross-sectional design, which precludes causal inference. The relatively small sample size (*n* = 41), together with adjustment for multiple covariates in the regression models, may have limited the statistical power and model stability to detect modest associations between tissue metal concentrations and global DNA methylation. Accordingly, the absence of statistically significant associations should be interpreted cautiously and does not necessarily exclude potential epigenetic effects of tissue metal accumulation. Tissue metal concentrations may also not reflect exposure during etiologically relevant windows of colorectal carcinogenesis. In addition, tissue metal concentrations were expressed on a wet-weight basis and were not normalized to dry weight or protein content, which may influence comparisons between tumor and adjacent tissues that differ in cellular composition and water content. Because analyses were performed on small wet tissue aliquots, the measured concentrations may also have been influenced by local tissue heterogeneity. Moreover, adjacent tissue used as a reference may not represent completely normal mucosa due to possible field cancerization effects. In addition, metals were measured only in colon tissue, and concurrent assessment of metal concentrations in blood or urine was not performed; therefore, relationships between tissue metal accumulation and systemic metal exposure could not be directly evaluated. Moreover, metals were quantified as total concentrations and metal speciation was not assessed. Because the biological activity and toxicity of some metals depend on their chemical form, future studies incorporating speciation analyses may provide additional mechanistic insight. Although certified reference material specific to colon tissue was not available during the study period, analytical performance was evaluated using matrix-matched spike recovery experiments and replicate precision analyses. For Hg in particular, the high proportion of concentrations below the MDL resulted in limited variability after MDL/2 substitution, which may have reduced the stability and interpretability of Hg-specific regression estimates. In addition, the comet assay was performed without positive and negative control materials, and only 25 nucleoids per sample were scored, which may have reduced analytical precision; however, all samples were processed and evaluated under standardized laboratory conditions to minimize technical variability. Furthermore, a range of lifestyle and environmental factors—such as smoking status, dietary habits, physical and occupational activities, housing environment, and psychosocial stress—are known to influence multiple (patho) physiological processes and may affect both metal exposure and epigenetic outcomes. Although information on some of these factors was collected, the limited sample size precluded their inclusion in multivariable analyses. While patients who had received chemotherapy were excluded to reduce potential treatment-related confounding, the influence of other medications prescribed for comorbid conditions cannot be entirely excluded and should be considered in future studies.

### Implications and future directions

Together, these findings suggest that while metals are detectable in colon tissue, their relationships with global epigenetic and genotoxic biomarkers are complex and not readily captured by circulating measures in established colon cancer. This observation highlights an important challenge in environmental epidemiology, where peripheral blood biomarkers are often used as proxies for tissue-specific biological effects. The limited concordance observed between tissue metal concentrations and blood-based biomarkers in the present study suggests that circulating markers may not adequately reflect localized molecular processes occurring within the tumor microenvironment. Future studies integrating tissue-based metal measurements with concurrent assessment of systemic exposure matrices in larger, longitudinal studies, along with high-resolution, locus-specific epigenomic profiling, may help better characterize tissue-specific and mixture-related effects of metal exposure on colorectal carcinogenesis. In addition, high-resolution epigenomic approaches, including locus-specific or genome-wide methylation profiling, may provide greater sensitivity for detecting exposure-related epigenetic alterations than global methylation measures alone (56, 58).

## Conclusion

In this cross-sectional study of colon cancer patients, metal concentrations in paired cancerous and non-cancerous colon tissues were not consistently associated with global DNA methylation in either tissue or peripheral blood. Apart from a significant reduction in Cd levels in cancerous tissue and an isolated inverse association between Hg in tumor tissue and systemic oxidative DNA damage, which should be interpreted with caution, most tissue metals showed limited relationships with epigenetic and genotoxic biomarkers. The weak concordance between tissue-based and blood-based measures highlights the tissue specificity of epigenetic regulation and underscores the limitations of circulating biomarkers in reflecting localized biological processes in the colon. Collectively, these findings suggest that global DNA methylation may be insufficient to capture subtle, metal-related epigenetic alterations in established colorectal cancer and emphasize the need for larger, longitudinal studies incorporating high-resolution, locus-specific epigenomic approaches to better elucidate the role of metal exposure in colorectal carcinogenesis.

## Data Availability

The original contributions presented in the study are included in the article/Supplementary material, further inquiries can be directed to the corresponding author.

## References

[1] Sung H Ferlay J Siegel RL Laversanne M Soerjomataram I Jemal A . Global cancer statistics 2020: GLOBOCAN estimates of incidence and mortality worldwide for 36 cancers in 185 countries. CA Cancer J Clin. (2021) 71:209–49. eng. doi: 10.3322/caac.21660 33538338

[2] Saudi Cancer Registry. Cancer Incidence Report Saudi Arabia 2015. Riyadh: Saudi Health Council, National Health Information Center (2018).

[3] Alsanea N Abduljabbar AS Alhomoud S Ashari LH Hibbert D Bazarbashi S. Colorectal cancer in Saudi Arabia: incidence, survival, demographics and implications for national policies. Ann Saudi Med. (2015) 35:196–202. eng. doi: 10.5144/0256-4947.2015.196 26409793 PMC6074461

[4] Murphy N Moreno V Hughes DJ Vodicka L Vodicka P Aglago EK. Lifestyle and dietary environmental factors in colorectal cancer susceptibility. Mol Aspects Med. (2019) 69:2–9. doi: 10.1016/j.mam.2019.06.005 31233770

[5] Thanikachalam K Khan G. Colorectal cancer and nutrition. Nutrients. (2019) 11:164. doi: 10.3390/nu11010164 30646512 PMC6357054

[6] Smith HG Nilsson PJ Shogan BD Harji D Gambacorta MA Romano A . Neoadjuvant treatment of colorectal cancer: comprehensive review. BJS Open. (2024) 8:zrae038. doi: 10.1093/bjsopen/zrae038 38747103 PMC11094476

[7] National Research Council. Diet, Nutrition, and Cancer: Directions for Research. Washington, DC: National Academies Press (1983). 25032418

[8] Rai PK Lee SS Zhang M Tsang YF Kim KH. Heavy metals in food crops: health risks, fate, mechanisms, and management. Environ Int. (2019) 125:365–85. doi: 10.1016/j.envint.2019.01.067 30743144

[9] Agency for Toxic Substances and Disease Registry (ATSDR). Substance Priority List. Atlanta, GA: U.S. Department of Health and Human Services, Public Health Service (2022).

[10] Bonfiglio R Sisto R Casciardi S Palumbo V Scioli MP Palumbo A . The impact of toxic metal bioaccumulation on colorectal cancer: unravelling the unexplored connection. Sci Total Environ. (2024) 906:167667. doi: 10.1016/j.scitotenv.2023.167667 37813250

[11] Zhu Y Costa M. Metals and molecular carcinogenesis. Carcinogenesis. (2020) 41:1161–72. doi: 10.1093/carcin/bgaa076 32674145 PMC7513952

[12] Hartwig A Schwerdtle T. Interactions by carcinogenic metal compounds with DNA repair processes: toxicological implications. Toxicol Lett. (2002) 127:47–54. doi: 10.1016/S0378-4274(01)00482-9 12052640

[13] Kopp B Zalko D Audebert M. Genotoxicity of 11 heavy metals detected as food contaminants in two human cell lines. Environ Mol Mutagen. (2018) 59:202–10. doi: 10.1002/em.22157 29150881

[14] Sohrabi M Gholami A Azar MH Yaghoobi M Shahi MM Shirmardi S . Trace element and heavy metal levels in colorectal cancer: comparison between cancerous and non-cancerous tissues. Biol Trace Elem Res. (2018) 183:1–8. doi: 10.1007/s12011-017-1099-7 28795369

[15] Alimonti A Bocca B Lamazza A Forte G Rahimi S Mattei D . A study on metals content in patients with colorectal polyps. J Toxicol Environ Health A. (2008) 71:342–7. doi: 10.1080/15287390701839133 18214808

[16] Urrutia-Ortega IM Garduno-Balderas LG Delgado-Buenrostro NL Freyre-Fonseca V Flores-Flores JO Gonzalez-Robles A . Food-grade titanium dioxide exposure exacerbates tumor formation in colitis associated cancer model. Food Chem Toxicol. (2016) 93:20–31. doi: 10.1016/j.fct.2016.04.014 27117919

[17] Proquin H Jetten MJ Jonkhout MCM Garduno-Balderas LG Briede JJ de Kok TM . Transcriptomics analysis reveals new insights in E171-induced molecular alterations in a mouse model of colon cancer. Sci Rep. (2018) 8:9738. doi: 10.1038/s41598-018-28063-z 29950665 PMC6021444

[18] Weir A Westerhoff P Fabricius L Hristovski K von Goetz N. Titanium dioxide nanoparticles in food and personal care products. Environ Sci Technol. (2012) 46:2242–50. doi: 10.1021/es204168d 22260395 PMC3288463

[19] Bettini S Boutet-Robinet E Cartier C Comera C Gaultier E Dupuy J . Food-grade TiO2 impairs intestinal and systemic immune homeostasis, initiates preneoplastic lesions and promotes aberrant crypt development in the rat colon. Sci Rep. (2017) 7:40373. doi: 10.1038/srep40373 28106049 PMC5247795

[20] Kiani B Hashemi Amin F Bagheri N Bergquist R Mohammadi AA Yousefi M . Association between heavy metals and colon cancer: an ecological study based on geographical information systems in North-Eastern Iran. BMC Cancer. (2021) 21:414. doi: 10.1186/s12885-021-08148-1 33858386 PMC8048218

[21] Jan AT Azam M Siddiqui K Ali A Choi I Haq QM. Heavy metals and human health: mechanistic insight into toxicity and counter defense system of antioxidants. Int J Mol Sci. (2015) 16:29592–630. doi: 10.3390/ijms161226183 26690422 PMC4691126

[22] Kruk J Aboul-Enein HY. Reactive oxygen and nitrogen species in carcinogenesis: implications of oxidative stress on the progression and development of several cancer types. Mini Rev Med Chem. (2017) 17:904–19. doi: 10.2174/1389557517666170228115324 28245782

[23] Ercal N Gurer-Orhan H Aykin-Burns N. Toxic metals and oxidative stress part I: mechanisms involved in metal-induced oxidative damage. Curr Top Med Chem. (2001) 1:529–39. doi: 10.2174/1568026013394831 11895129

[24] Valko M Rhodes CJ Moncol J Izakovic M Mazur M. Free radicals, metals and antioxidants in oxidative stress-induced cancer. Chem Biol Interact. (2006) 160:1–40. doi: 10.1016/j.cbi.2005.12.009 16430879

[25] Shoeb M Zarus GM Abadin HE. Profiling metal-induced genotoxic endpoints. J Environ Health. (2023) 86:30–5. 39239121 PMC11375607

[26] Gao X Holleczek B Cuk K Zhang Y Anusruti A Xuan Y . Investigation on potential associations of oxidatively generated DNA/RNA damage with lung, colorectal, breast, prostate and total cancer incidence. Sci Rep. (2019) 9:7109. doi: 10.1038/s41598-019-42596-x 31068619 PMC6506483

[27] Guo C Li X Wang R Yu J Ye M Mao L . association between oxidative dna damage and risk of colorectal cancer: sensitive determination of urinary 8-Hydroxy2′-deoxyguanosine by UPLC-MS/MS analysis. Sci Rep. (2016) 6:32581. doi: 10.1038/srep32581 27585556 PMC5009303

[28] Singh NP McCoy MT Tice RR Schneider EL. A simple technique for quantitation of low levels of DNA damage in individual cells. Exp Cell Res. (1988) 175:184–91. doi: 10.1016/0014-4827(88)90265-0 3345800

[29] Goel A Boland CR. Epigenetics of colorectal cancer. Gastroenterology. (2012) 143:1442–60. doi: 10.1053/j.gastro.2012.09.032 23000599 PMC3611241

[30] Sakai E Nakajima A Kaneda A. Accumulation of aberrant DNA methylation during colorectal cancer development. World J Gastroenterol. (2014) 20:978–87. doi: 10.3748/wjg.v20.i4.978 24574770 PMC3921549

[31] Chen J Sun H Tang W Zhou L Xie X Qu Z . DNA methylation biomarkers in stool for early screening of colorectal cancer. J Cancer. (2019) 10:5264–71. doi: 10.7150/jca.34944 31602277 PMC6775613

[32] Dong L Ren H. Blood-based DNA methylation biomarkers for early detection of colorectal cancer. J Proteomics Bioinform. (2018) 11:120–6. doi: 10.4172/jpb.1000477 30034186 PMC6054487

[33] Barrow TM Klett H Toth R Bohm J Gigic B Habermann N . Smoking is associated with hypermethylation of the APC 1A promoter in colorectal cancer: the ColoCare Study. J Pathol. (2017) 243:366–75. doi: 10.1002/path.4955 28791728 PMC5647242

[34] Nystrom M Mutanen M. Diet and epigenetics in colon cancer. World J Gastroenterol. (2009) 15:257–63. doi: 10.3748/wjg.15.257 19140224 PMC2653321

[35] Varela-Rey M Woodhoo A Martinez-Chantar ML Mato JM Lu SC. Alcohol, DNA methylation, and cancer. Alcohol Res. (2013) 35:25–35. doi: 10.35946/arcr.v35.1.04 24313162 PMC3860423

[36] Maleknia M Ahmadirad N Golab F Katebi Y Haj Mohamad Ebrahim Ketabforoush A DNA. Methylation in cancer: epigenetic view of dietary and lifestyle factors. Epigenetics Insights. (2023) 16:25168657231199893. doi: 10.1177/25168657231199893 37720354 PMC10504848

[37] Nassar FJ Msheik ZS Nasr RR Temraz SN. Methylated circulating tumor DNA as a biomarker for colorectal cancer diagnosis, prognosis, and prediction. Clin Epigenetics. (2021) 13:111. doi: 10.1186/s13148-021-01095-5 34001239 PMC8130320

[38] Ladeira C Koppen G Scavone F Giovannelli L. The comet assay for human biomonitoring: effect of cryopreservation on DNA damage in different blood cell preparations. Mutat Res Genet Toxicol Environ Mutagen. (2019) 843:11–7. doi: 10.1016/j.mrgentox.2019.02.002 31421731

[39] Møller P Azqueta A Boutet-Robinet E Koppen G Bonassi S Milić M . Minimum information for reporting on the comet assay (MIRCA): recommendations for describing comet assay procedures and results. Nat Protoc. (2020) 15:3817–26. doi: 10.1038/s41596-020-0398-1 33106678 PMC7688437

[40] Lee E Oh E Lee J Sul D Lee J. Use of the tail moment of the lymphocytes to evaluate DNA damage in human biomonitoring studies. Toxicol Sci. (2004) 81:121–32. doi: 10.1093/toxsci/kfh184 15178808

[41] Lucena-Aguilar G Sánchez-López AM Barberán-Aceituno C Carrillo-Ávila JA López-Guerrero JA Aguilar-Quesada R . Source selection for downstream applications based on DNA quality indicators analysis. Biopreserv Biobank. (2016) 14:264–70. doi: 10.1089/bio.2015.0064 27158753 PMC4991598

[42] Zeraatkar D Cheung K Milio K Zworth M Gupta A Bhasin A . Methods for the selection of covariates in nutritional epidemiology studies: a meta-epidemiological review. Curr Dev Nutr. (2019) 3:nzz104. doi: 10.1093/cdn/nzz104 31598577 PMC6778415

[43] Vander Heiden MG Cantley LC Thompson CB. Understanding the warburg effect: the metabolic requirements of cell proliferation. Science. (2009) 324:1029–33. doi: 10.1126/science.1160809 19460998 PMC2849637

[44] Juloski JT Rakic A Cuk VV Cuk VM Stefanović S Nikolić D . Colorectal cancer and trace elements alteration. J Trace Elem Med Biol. (2020) 59:126451. doi: 10.1016/j.jtemb.2020.126451 31954212

[45] Klimczak M Dziki A Kilanowicz A Sapota A Duda-Szymańska J Daragó A. Concentrations of cadmium and selected essential elements in malignant large intestine tissue. Prz Gastroenterol. (2016) 11:24–9. doi: 10.5114/pg.2015.52563 27110307 PMC4814536

[46] Dharmasivam M Kaya B. Transmetalation in cancer pharmacology. Int J Mol Sci. (2025) 26:11008. doi: 10.3390/ijms262211008 41303491 PMC12652228

[47] Ramón YCS Sesé M Capdevila C Aasen T De Mattos-Arruda L Diaz-Cano SJ . Clinical implications of intratumor heterogeneity: challenges and opportunities. J Mol Med (Berl). (2020) 98:161–77. doi: 10.1007/s00109-020-01874-2 31970428 PMC7007907

[48] Jomova K Alomar SY Valko R Fresser L Nepovimova E Kuca K . Interplay of oxidative stress and antioxidant mechanisms in cancer development and progression. Arch Toxicol. (2025). doi: 10.1007/s00204-025-04146-5 40906205 PMC12858508

[49] Iyengar V Woittiez J. Trace elements in human clinical specimens: evaluation of literature data to identify reference values. Clin Chem. (1988) 34:474–81. doi: 10.1093/clinchem/34.3.474 3280162

[50] Yaman M Kaya G Yekeler H. Distribution of trace metal concentrations in paired cancerous and non-cancerous human stomach tissues. World J Gastroenterol. (2007) 13:612–8. doi: 10.3748/wjg.v13.i4.612 17278230 PMC4065986

[51] Baba A Câtoi C. TUMOR CELL MORPHOLOGY. Comparative Oncology. Bucharest (RO): The Publishing House of the Romanian Academy (2007). Chapter 3. Available online at: https://www.ncbi.nlm.nih.gov/books/NBK9553/20806453

[52] Squitti R Pal A Dhar A Shamim MA Goswami K De Luca A . The debated issue on tissue copper levels in colorectal cancer patients: a meta-analysis and replication study. Biol Trace Elem Res. (2025) 203:3479–93. doi: 10.1007/s12011-024-04421-z 39433591

[53] Dakubo GD Jakupciak JP Birch-Machin MA Parr RL. Clinical implications and utility of field cancerization. Cancer Cell Int. (2007) 7:2. doi: 10.1186/1475-2867-7-2 17362521 PMC1838897

[54] Luo Y Yu M Grady WM. Field cancerization in the colon: a role for aberrant DNA methylation? Gastroenterol Rep. (2014) 2:16–20. doi: 10.1093/gastro/got039 24760232 PMC3920999

[55] Weyde KVF Olsen A-K Duale N Kamstra JH Skogheim TS Caspersen IH . Gestational blood levels of toxic metal and essential element mixtures and associations with global DNA methylation in pregnant women and their infants. Sci Total Environ. (2021) 787:147621. doi: 10.1016/j.scitotenv.2021.147621 34000534

[56] Elkin ER Higgins C Aung MT Bakulski KM. Metals exposures and DNA methylation: current evidence and future directions. Curr Environ Health Rep. (2022) 9:673–96. doi: 10.1007/s40572-022-00382-4 36282474 PMC10082670

[57] Lakshminarasimhan R Liang G. The role of DNA methylation in cancer. Adv Exp Med Biol. (2016) 945:151–72. doi: 10.1007/978-3-319-43624-1_7 27826838 PMC7409375

[58] Manić L Wallace D Onganer PU Taalab YM Farooqi AA Antonijević B . Epigenetic mechanisms in metal carcinogenesis. Toxicol Rep. (2022) 9:778–87. doi: 10.1016/j.toxrep.2022.03.037 36561948 PMC9764177

[59] Wallace MA Kormos TM Pleil JD. Blood-borne biomarkers and bioindicators for linking exposure to health effects in environmental health science. J Toxicol Environ Health B Crit Rev. (2016) 19:380–409. doi: 10.1080/10937404.2016.1215772 27759495 PMC6147038

[60] Ladeira C Møller P Giovannelli L Gajski G Haveric A Bankoglu EE . The comet assay as a tool in human biomonitoring studies of environmental and occupational exposure to chemicals-a systematic scoping review. Toxics. (2024) 12:270. doi: 10.3390/toxics12040270 38668493 PMC11054096

[61] Møller P Bankoglu EE Stopper H Gajski G Gerić M Haveric A . The comet assay as a tool in human biomonitoring of exposure to heavy metals – A systematic review and meta-analysis. Mutat Res Rev Mutat Res. (2025) 796:108567. doi: 10.1016/j.mrrev.2025.108567 41223799

[62] Shoeb M Kodali VK Farris BY Bishop LM Meighan TG Salmen R . Oxidative stress, DNA methylation, and telomere length changes in peripheral blood mononuclear cells after pulmonary exposure to metal-rich welding nanoparticles. NanoImpact. (2017) 5:61–9. doi: 10.1016/j.impact.2017.01.001 30734006 PMC6363128

[63] Shoeb M Mustafa GM Kodali VK Smith K Roach KA Boyce G . A possible relationship between telomere length and markers of neurodegeneration in rat brain after welding fume inhalation exposure. Environ Res. (2020) 180:108900. doi: 10.1016/j.envres.2019.108900 31711660 PMC6899181

[64] Shoeb M Meighan T Kodali VK Abadin H Faroon O Zarus GM . TERT-independent telomere elongation and shelterin dysregulation after pulmonary exposure to stainless-steel welding fume *in-vivo*. Environ Res. (2024) 250:118515. doi: 10.1016/j.envres.2024.118515 38373547 PMC11375608

[65] Sánchez-Alarcón J Milić M Bustamante-Montes LP Isaac-Olivé K Valencia-Quintana R Ramírez-Durán N. Genotoxicity of mercury and its derivatives demonstrated *in vitro* and *in vivo* in human populations studies. Syst Rev Toxics. (2021) 9:326. doi: 10.3390/toxics9120326 PMC8704886 34941760

[66] McKay JA Xie L Harris S Wong YK Ford D Mathers JC. Blood as a surrogate marker for tissue-specific DNA methylation and changes due to folate depletion in post-partum female mice. Mol Nutr Food Res. (2011) 55:1026–35. doi: 10.1002/mnfr.201100008 21520493

[67] Edgar RD Jones MJ Meaney MJ Turecki G Kobor MS. BECon: a tool for interpreting DNA methylation findings from blood in the context of brain. Transl Psychiatry. (2017) 7:e1187–e1187. doi: 10.1038/tp.2017.171 28763057 PMC5611738

[68] Li L Choi JY Lee KM Sung H Park SK Oze I . DNA methylation in peripheral blood: a potential biomarker for cancer molecular epidemiology. J Epidemiol. (2012) 22:384–94. doi: 10.2188/jea.JE20120003 22863985 PMC3798632

[69] Chilimoniuk Z Gładysz K Moniczewska N Chawrylak K Pelc Z Mlak R. The role of epigenetic biomarkers as diagnostic, predictive and prognostic factors in colorectal cancer. Cancers. (2025) 17:2632. doi: 10.3390/cancers17162632 40867261 PMC12384644

