# Supplementary material for: Metal accumulation in paired colon cancer and adjacent tissues and its relationship with genotoxic and epigenetic biomarkers
Source: Front Public Health. 2026 Apr 14;14:1801314. doi: 10.3389/fpubh.2026.1801314 (PMC13121328; doi:10.3389/fpubh.2026.1801314)
Supplement: Supplementary file 1 [file Data_Sheet_1.docx]

**Supplementary Material**

**Metal Accumulation in Paired Colon Cancer and Adjacent Tissues and Its Relationship with Genotoxic and Epigenetic Biomarkers**

Iman Al-Saleh^a*^, Ghofran Al-Qudaihi^a^, Reem Al-Rouqi^a^, Nujud Alrushud^a^, Hissah Alnuwaysir^a^, Samar Alhomoud^b^, Alaa Abdul Jabbar^b^, Luai Ashari^b^, Raha Alahmadi^b^, Hussain Alkhdhur^b^, Salah Addin Falah^b^, Hadeel Almanea^c^, Hussah Alhussaini^c^, Anmar Semilan^b^, Rasha Althebaity^b^, Saud Alharbi^a^

^a^ Environmental Health Program

^b^ Surgical Oncology Department

^c^ Anatomic Pathology Department

King Faisal Specialist Hospital and Research Centre, Riyadh, Saudi Arabia

**^a^ Corresponding Author Address:**

Dr. Iman Al-Saleh (MBC#03)

Environmental Health Program

King Faisal Specialist Hospital & Research Centre

P.O. Box: 3354, Riyadh 11211, Saudi Arabia

E-mail: [iman@kfshrc.edu.sa](mailto:iman@kfshrc.edu.sa)

**Table S1**. Detection frequency of metals in colon tissues

| **Metal** | **MDL (µg/L)** | **% > MDL (non-cancerous tissue)** | **% > MDL (cancerous tissue)** |
| --- | --- | --- | --- |
| Pb | 0.017 | 74% | 84% |
| Cd | 0.0005 | 92% | 82% |
| Hg | 0.073 | 16% | 12% |
| As | 0.002 | 38% | 42% |
| Cr | 0.131 | 94% | 94% |
| Ti | 0.039 | 96% | 94% |

**Table S2**. Associations between tissue metals and blood DNA damage – %DNA (adjusted models, n = 41)

| **Metal** | **β** | **Non-cancerous tissue** | | |  | **Cancerous tissue** | | |
| --- | --- | --- | --- | --- | --- | --- | --- | --- |
|  |  | **95% CI (L)** | **95% CI (U)** | **p** | **β** | **95% CI (L)** | **95% CI (U)** | **p** |
| Pb | 0.001 | -0.082 | 0.083 | 0.989 | 0.023 | -0.080 | 0.126 | 0.655 |
| Cd | 0.027 | -0.077 | 0.130 | 0.602 | 0.004 | -0.083 | 0.092 | 0.918 |
| Hg | -0.168 | -0.498 | 0.163 | 0.31 | -0.470 | -0.825 | -0.115 | **0.011** |
| As | 0.009 | -0.072 | 0.09 | 0.825 | -0.003 | -0.078 | 0.073 | 0.942 |
| Cr | -0.006 | -0.199 | 0.187 | 0.952 | 0.082 | -0.076 | 0.241 | 0.299 |
| Ti | 0.023 | -0.145 | 0.190 | 0.784 | -0.020 | -0.143 | 0.102 | 0.737 |
| Metal Burden Index | -0.013 | -0.503 | 0.478 | 0.958 | -0.087 | -0.562 | 0.389 | 0.713 |

Models adjusted for age, sex, BMI, educational attainment, and tumor location. Metal concentrations were log-transformed before analysis. β represents the unstandardized regression coefficient.
